# Supplementary material for: Identification of Homologous Polyprenols from Thermophilic Bacteria
Source: Microorganisms. 2021 May 28;9(6):1168. doi: 10.3390/microorganisms9061168 (PMC8226974; doi:10.3390/microorganisms9061168)
Supplement: Supplementary file 1 [file microorganisms-09-01168-s001.zip › microorganisms-1222060-supplementary.pdf]

Supplementary Materials

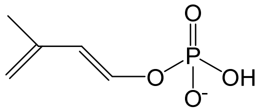

Figure S1. The structure of the ion at *m/z* 163.0165 ($C_5H_8O_4P^-$)

Table S1. Chemical composition of the spring "Vřídlo".

Chemical composition[a]	mg/L[b]
Na	1600
K	99
Li	2.9
NH_4	0.36
Ca	127
Mg	44
Sr	2
Ba	0.05
Fe	1.03
Mn	0.16
Cl	613
Br	1.6
I	0.021
F	6.2
HCO_3	2110
NO_3	0.00
SO_4	1400
SiO_2	71
CO_2	380

[a] Reference [1] [b] traces, i.e. less than 0.05 mg/L – Cu, Be, Zn, UO_2, HS, NO_2, HPO_4, $HAsO_2$.

Table S2. Designation of compounds based on their length and number of carbon in the chain, detected and theoretical masses of ions $[M-H]^-$ and/or $[M+Li]^+$ (Da), and molecular formulas.

Compound	Detected mass [*m/z*]	Theoretical mass [Da]	Molecular formula
C55	765.6917	765.6919	$C_{55}H_{89}O^-$
C55-P	845.6580	845.6582	$C_{55}H_{90}O_4P^-$
C55-PP	925.6244	925.6246	$C_{55}H_{91}O_7P_2^-$
C40	569.5266	569.5268	$C_{40}H_{66}LiO^+$
C45	637.5891	637.5894	$C_{45}H_{74}LiO^+$
C50	705.6518	705.6520	$C_{50}H_{82}LiO^+$
C55	773.7144	773.7146	$C_{55}H_{90}LiO^+$
C60	841.7770	841.7772	$C_{60}H_{98}LiO^+$
C65	909.8395	909.8398	$C_{65}H_{106}LiO^+$

Table S3. The data of tandem mass spectrum of C55-PP.

m/z	**Abundance (%)**
158.9252	90.5
160.9409	4.3
162.9563	4.0
164.9720	2.2
176.9356	91.8
177.1641	0.6
178.9507	2.8
190.9512	0.8
245.2271	0.6
585.5402	0.6
653.6028	0.6
719.6500	0.8
721.6653	1.8
723.6811	1.6
733.6653	3.1
745.6651	1.5
747.6810	2.0
763.6761	2.1
765.6915	4.8
825.6315	2.0
827.6471	3.6
843.6420	0.8
845.6581	6.2
883.5769	1.4
885.5930	1.6
889.6030	1.1
907.6133	19.4
925.6242	100.0
